# Genetic Diversity Analysis of *Dociostaurus maroccanus* (Thunberg, 1815) (Orthoptera: Acrididae), a Newly Recorded Species, Based on Combined *COI* and *Cytb* Mitochondrial Gene Markers

**DOI:** 10.3390/insects17070726

**Published:** 2026-07-14

**Authors:** Shiying He, Huixia Liu, Xudong Zha, Rong Ji, Zhong Liang, Roman Jashenko, Yongjun Zhang, Lan He

**Affiliations:** 1 International Research Center of Cross-Border Pest Management in Central Asia, Xinjiang Key Laboratory of Special Species Conservation and Regulatory Biology, College of Life Sciences, Xinjiang Normal University, Urumqi 830017, China; 2 Tacheng, Research Field (Migratory Biology), Observation and Research Station of Xinjiang, Tacheng 834700, China; 3 Changji University Office, Changji 831100, China; 4 Xinjiang Uygur Autonomous Region Grassland Biological Disaster Prevention and Control Center, Urumqi 830017, China; 5 Institute of Zoology, Ministry of Education and Science of Kazakhstan, Almaty 050038, Kazakhstan

**Keywords:** *Dociostaurus maroccanus*, mitochondrial DNA, geographical populations, China–Kazakhstan border

## Abstract

*Dociostaurus maroccanus* is a major migratory pest first recorded in Xinjiang, China, in 2025 in the Ili River Valley and Tacheng City. This event represents a new situation of concern in the field of biological security along China’s northwestern border that warrants attention. This study investigated the genetic diversity and population structure of four populations from the China–Kazakhstan border region using combined mitochondrial cytochrome C oxidase subunit I (*COI*) and cytochrome B (*Cytb*) gene markers. A total of 41 haplotypes were identified from 74 individuals, with the shared haplotypes H3 and H6 being the most frequent. Haplotype network analysis revealed no clear geographic population structuring. Haplotype diversity was high (*H*d = 0.957), whereas nucleotide diversity was low (*π* = 0.00212), indicating low overall genetic diversity. Frequent gene flow (*N*m = 12.31–43.08) and minimal genetic differentiation (*F*st = −0.0255 to 0.0199) were detected among populations. AMOVA indicated that genetic variation mainly occurred within populations (100.12%), with no significant differentiation among populations (−0.59%). Neutrality tests (Tajima’s D = −2.45136, *p* < 0.05; Fu’s Fs = −44.037, *p* < 0.001), the unimodal mismatch distribution, and goodness-of-fit test results for KZ1 population (SSD = 0.0028, *p* = 0.91; raggedness = 0.0225, *p* = 0.86), KZ2 population (SSD = 0.00846, *p* = 0.25; raggedness = 0.0650, *p* = 0.13) and YN population further support the expansion phenomenon. Mantel test (*r* = −0.5948, *p* = 0.9583) preliminarily indicated that population distribution was not correlated with geographic distance. Therefore, in the region covered by this study, the overall characteristics show recent population expansion features. The preliminary results of this study provide basic materials for understanding the genetic background of this species in China’s border regions and can serve as a reference for subsequent population dynamic monitoring and control work.

## Simple Summary

In this study, *Dociostaurus maroccanus* individuals were collected from four geographic populations along the China–Kazakhstan border. Using combined mitochondrial DNA markers from the cytochrome C oxidase subunit I (*COI*) and cytochrome B (*Cytb*) gene regions, we analyzed the genetic diversity and population structure of these populations. Results indicate gene flow among the four populations, with no significant genetic differentiation detected. Neutrality tests and unimodal mismatch distribution analyses showed that various populations may have experienced recent population expansion signals, with the goodness-of-fit test results for mismatch distribution in KZ1, KZ2, and YN populations further supporting the existence of expansion signals. The observed combination of low genetic diversity, extensive gene flow, and detected expansion signals preliminarily indicates the possibility of recent dispersal of this species in the China–Kazakhstan border region. This study provides baseline reference information for understanding the genetic background of this newly recorded *D. maroccanus* population in China.

## 1. Introduction

*Dociostaurus maroccanus* (Thunberg, 1815) (Orthoptera: Acrididae) is widely distributed in arid and semi-arid regions from the Mediterranean coast to Central Asia. This species is characterized by polyphagy, high reproductive capacity, and strong migratory ability. With a host range spanning 33 families of economically important crops, including wheat, maize, and cotton, this species poses a serious threat to both agricultural production and ecosystem stability [1,2,3,4]. Adults can migrate over distances of 70–100 km, with some individuals capable of dispersing up to 200 km. During outbreak periods, swarming populations can cause severe damage to crops and grasslands, resulting in substantial economic losses [1,3,5]. For many years, no official occurrence records of *D. maroccanus* were reported in China [6]. However, in June 2025, this species was first detected and identified as a new Chinese record species in the Ili River Valley and TaCheng (TC) City of Xinjiang, China [7,8]. It has been confirmed as an invasive foreign species by the State Forestry and Grassland Administration of China [9], which has triggered high concern about biosecurity along China’s northwestern border. Regardless of whether the origin of this species stems from recent cross-border dispersal or natural range expansion, its sudden emergence poses a potential challenge to biosecurity along the northwestern border, and it is urgently needed to clarify this issue from the population genetics perspective.

Previous studies on *D. maroccanus* have mainly focused on its population dynamics [1,10,11], biological characteristics [2,12,13,14], and control strategies [15,16]. Regarding population genetics, González-Serna et al. analyzed populations from the Iberian Peninsula and the Canary Islands using high-density SNP markers and revealed distinct regional genetic patterns and demographic histories [17]. However, the aforementioned genetic studies have primarily concentrated on Europe and North Africa, whereas the population genetic characteristics of Central Asian populations, particularly those in Kazakhstan (KZ) bordering Xinjiang, China, remain poorly understood. Moreover, as a newly recorded species in China [7,8,18], *D. maroccanus* has a short recorded period in this region, and its genetic origins of the established population and phylogenetic relationships with populations outside China remain unclear, with multiple plausible explanations existing. Therefore, investigating its population genetic structure at a transboundary regional scale is important for exploring multiple potential diffusion scenarios and evaluating potential dispersal risks.

Population genetic structure and diversity are key indicators for understanding the dispersal dynamics and adaptive evolution of invasive species. They also play a crucial role in predicting expansion trends [19]. Due to their maternal inheritance and moderate evolutionary rates, mitochondrial genes are widely used for tracing origins and assessing population differentiation in insects [20,21]. Among mitochondrial markers, the cytochrome C oxidase subunit I (*COI*) gene has been extensively applied in species identification, population dynamics, dispersal analysis, and phylogenetic reconstruction, demonstrating its versatility beyond taxonomy. For example, Yuan et al. analyzed the *COI* gene of *Bactrocera dorsalis* from 15 geographic populations in China and found high levels of polymorphism and haplotype diversity, suggesting a northward expansion from Hainan, Guangxi, and Guangdong provinces [22]. Similarly, Yuan et al. investigated the genetic differentiation of *Tuta absoluta* populations in China and reported significant divergence at the mitochondrial DNA level between populations from Gansu and Ningxia [23]. However, analyses based on a single mitochondrial marker, as a single non-recombining genetic unit, may not fully capture the complexity of population history. Nevertheless, mtDNA, due to its relatively fast evolutionary rate and mature application, still provides a basic reference for species identification and maternal lineage inference. Future studies integration of nuclear genomic loci will further supplement more complete dynamics of population history.

The countries neighboring China, including Kazakhstan, Kyrgyzstan, and Tajikistan, have records of *D. maroccanus* occurrence [1,3]. Xinjiang borders directly with Kazakhstan and shares similar grassland ecosystems and climatic conditions with these neighboring regions, with few natural geographical barriers restricting insect dispersal, making it an important potential channel for transboundary locust migration [24,25,26]. Although current research has preliminarily speculated that the species may originate from Almaty Region in Kazakhstan [27], this hypothesis requires further verification due to limited sample data from other neighboring countries. In the present study, we targeted *D. maroccanus* occurrence areas in Yining and Tacheng cities, as well as the adjacent Almaty Region, identifying and collecting samples from a total of 4 occurrence sites. Using combined mitochondrial *COI* and cytochrome B (*Cytb*) gene sequences, we systematically analyzed the genetic diversity, population structure, and gene flow of 4 populations along the China–Kazakhstan border. This study aims to evaluate gene flow patterns among populations and explore multiple possible scenarios of *D. maroccanus* in Xinjiang, China, clarifying its population genetic characteristics, and providing a basic reference for source tracing and precise prevention and control of *D. maroccanus*. The research findings will provide a scientific basis for early warning, cross-border tracing, and precise prevention and control of *D. maroccanus*, and have important theoretical value and practical significance for protecting agricultural production and ecological security in northwestern China.

## 2. Materials and Methods

### 2.1. Tested Locust Sources and Their Geographic Distribution

**Figure 1:**
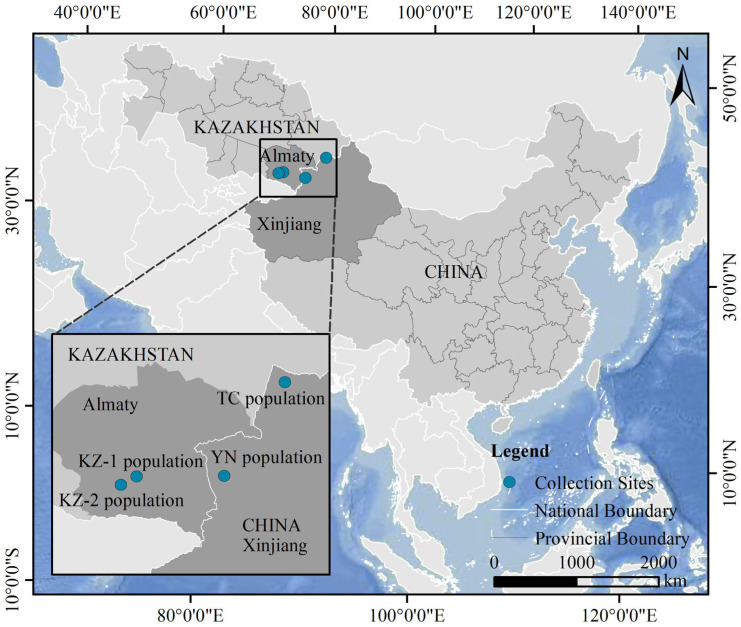
Location distribution of sampling points for *D. maroccanus*. Note: The white boundaries represent national borders, gray areas represent China and Kazakhstan respectively, dark gray areas indicate the sampling regions, and blue circles represent the sampling points.

*D*. *maroccanus* specimens examined in this study were collected from two sites in Yining and Tacheng, Xinjiang, China, and from Almaty Province, Kazakhstan. A total of 74 adult specimens were collected between June and July 2025. Detailed information on the collection sites is provided in Figure 1 and Table 1. All collected specimens were preserved in anhydrous ethanol and stored at −20 °C until analysis.

### 2.2. Genomic DNA Extraction

The hind femora of *D. maroccanus* specimens preserved in anhydrous ethanol were excised and rinsed with double-distilled water (ddH_2_O). After drying with absorbent paper, the tissues were transferred to 2 mL centrifuge tubes. Total genomic DNA was extracted according to the manufacturer’s instructions using a DNA Extraction Kit (DP304; Tiangen Biotechnology Co., Ltd., Beijing, China). DNA concentration and purity were determined using an ultra-micro spectrophotometer. The extracted DNA was stored at −20 °C until further analysis.

### 2.3. PCR Amplification and Sequencing

Total DNA was extracted and used as the template for amplification of mitochondrial *COI* and *Cytb* genes. Primer sequences were designed based on published mitochondrial sequences available in GenBank, yielding two pairs of gene-specific primers targeting the *COI* and *Cytb* loci. All primers were synthesized by Sangon Biotech Co., Ltd. (Shanghai, China).

The primer sequences were as follows:

*COI* forward: 5′-TAGCAGGAGCTATTGCTCATG-3′;

*COI* reverse: 5′-GTCGAGGTATTCCTGCTAATCC-3′;

*Cytb* forward: 5′-TCTAATTGACTTACCAGCACCAAC-3′;

*Cytb* reverse: 5′-GGTTCTTCAACTGGTCGTTTACCA-3′.

PCR was performed in a 25 μL reaction mixture containing 12.5 μL of 2× Taq PCR Premix (TIANGEN KT211-02) (Tiangen Biotechnology Co., Ltd., Beijing, China), 9.5 μL of ddH_2_O, 1 μL each of forward and reverse primers, and 1 μL of template DNA. A no-template control was included in each run to monitor contamination. PCR cycling conditions were as follows: initial denaturation at 95 °C for 3 min; 30 cycles of 95 °C for 45 s, 54 °C for 30 s, and 72 °C for 2 min; followed by a final extension at 72 °C for 10 min and holding at 4 °C. PCR products were stored at −20 °C until further analysis. Amplification products were analyzed by 1% agarose gel electrophoresis using a 2 μL DNA marker as a size standard. Bands were visualized using a gel imaging system. PCR products were purified and subjected to bidirectional sequencing by Sangon Biotech Co., Ltd., Shanghai, China.

### 2.4. Data Analysis

Sequencing chromatograms were analyzed using SnapGene 6.0.2, and the resulting sequences were manually edited in MEGA 12 before multiple sequence alignment. Sequence alignment analysis was verified by comparing with published *D. maroccanus* mitochondrial *COI* and *Cytb* gene sequences available in the National Center for Biotechnology Information (NCBI) database (GenBank accession numbers: KM384841.1 and DQ230814.4). The number of polymorphic sites (*S*), haplotype diversity (*H*d), nucleotide diversity (π), and the average number of nucleotide differences (*K*) were calculated using DnaSP v5 [28]. Tajima’s D and Fu’s Fs neutrality tests, together with analysis of molecular variance (AMOVA) and mismatch distribution analysis, were performed to evaluate genetic variation within and among populations. Genetic distances among populations were calculated in MEGA 7.0 using the Kimura 2-parameter (K2P) model [29]. Population genetic differentiation coefficient (*F*st), gene flow (*N*m) and mismatch distribution goodness-of-fit tests (SSD and Raggedness) were estimated using Arlequin v3.11 [30]. Haplotype networks were constructed using PopArt. Geographic distances between sampling sites were calculated from latitude and longitude coordinates [31]. The vegan package in R v4.5.2 was used to test the correlation between genetic and geographic distances among populations.

## 3. Results

Amplification of mitochondrial *COI* and *Cytb* gene fragments was successful for all 74 *D. maroccanus* samples. Sequence alignment using existing *D. maroccanus COI* and *cytb* sequences from GenBank (accession numbers: KM384841.1 and DQ230814.4) confirmed that all amplified sequences corresponded to the *COI* and *Cytb* genes of *D. maroccanus*. After excluding low-quality regions, a valid combined sequence of 1518 bp was obtained, with no insertions or deletions. The analysis identified 60 variable sites. The nucleotide composition of the combined sequence was A: 32.0%, G: 14.7%, C: 17.2%, and T: 36.1%, resulting in an A + T content of 68.1%, higher than the C + G content of 31.9%.

### 3.1. Haplotype Analysis

**Figure 2:**
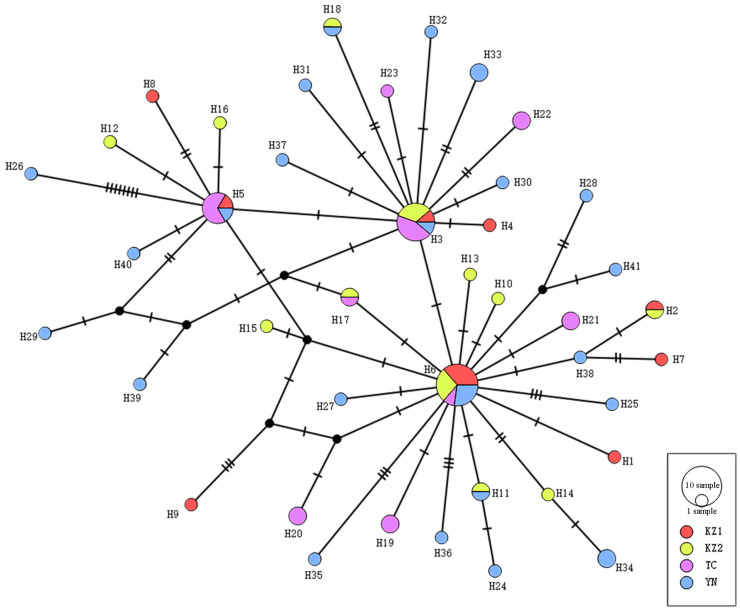
Haplotype network of combined mitochondrial *COI* and *Cytb* genes of *D. maroccanus* from different geographic populations along the China–Kazakhstan border. Note: In the haplotype network, each circle represents a unique haplotype, sized proportionally to the number of samples (1-10). Lines connecting circles show genetic/evolutionary relationships, with vertical bars indicating base substitutions. Four colors represent populations: KZ1 (red), KZ2 (yellow), TC (purple), YN (blue).

Analysis of the combined *COI* and *Cytb* sequences identified 41 haplotypes across the four geographic populations of *D. maroccanus*, including seven shared haplotypes and 34 population-specific haplotypes. Among the shared haplotypes, H3 and H6 were the most common, occurring in all four populations and represented by nine and 11 individuals, respectively. H5 was the next most common shared haplotype and was detected in six individuals from the KZ1 population in the Yenbekshy Kazakh District of Almaty Region, Kazakhstan, the YN population in Yining city, Xinjiang, China, and the TC population in Tacheng City, Xinjiang, China. The remaining four shared haplotypes (H2, H11, H17, H18) were less frequent, and each occurred in only two individuals from specific population pairs (H2: KZ1–KZ2 populations, with KZ2 population located in Ile District, Almaty Region, Kazakhstan; H11 and H18: KZ1–TC populations; H17: KZ2–TC populations). The distribution of population-specific haplotypes differed among populations. The YN population contained the largest number of unique haplotypes (H24–H41; *n* = 18), followed by KZ2 population (H10, H12–H16; *n* = 6), TC population (H19–H23; *n* = 5), and KZ1 population (H1, H4, H7–H9; *n* = 5). The vast majority of population-specific haplotypes were observed in only a single individual within their respective populations. The haplotype network exhibited a radiating structure, with no distinct clades corresponding to geographic populations (Figure 2).

### 3.2. Genetic Diversity Analysis

The results of the genetic diversity analyses are summarized in Table 2. Across all populations, 41 haplotypes (*h*) were detected, with haplotype diversity (*H*d) of 0.957, nucleotide diversity (*π*) of 0.00212, and an average number of nucleotide differences (*K*) of 3.22399. These values indicate high haplotype diversity but low nucleotide diversity. The number of haplotypes per population ranged from 9 to 23, haplotype diversity (*H*d) from 0.906 to 0.986, and nucleotide diversity (*π*) from 0.001 49 to 0.00291. Among these populations, the YN population exhibited the highest haplotype diversity, while nucleotide diversity was highest in the YN population and lowest in the TC population.

### 3.3. Genetic Differentiation Analysis

Pairwise *F*st and Nm analyses indicated extremely low levels of genetic differentiation among the four geographic populations of *D. maroccanus* (Table 3). *F*st values ranged from −0.02553 to 0.01990, all below the significant threshold of 0.05, with negative *F*st values observed in the KZ1–KZ2 (*F*st = −0.02553), KZ1–YN (*F*st = −0.00642), and KZ2–YN (*F*st = −0.01828) population pairs, while other population pairs exhibited Fst values approaching zero, indicating that no actual genetic differentiation between populations. Gene flow estimates (*N*m) ranged from 12.31 to 43.08 (with some comparisons marked as “N/A” due to negative Fst values leading to non-estimable *N*m), with the highest *N*m value of 43.08 observed between YN and TC populations, far exceeding the critical value of *N*m > 1 required to maintain population homogenization; some comparisons were marked as “N/A” due to negative *F*st values leading to non-estimable *N*m.

Table 4 presents the results of AMOVA analysis among populations. Based on geographic origin differences, the four populations were divided into two groups in the AMOVA analysis: Kazakhstani populations (KZ1 and KZ2) as one group and Chinese populations (YN and TC) as another group. This grouping strategy is based on the two geographic sources (Kazakhstan vs. China), consistent with our hypothesis to distinguish between potential source populations and newly established populations of this species. The results showed that genetic variation was primarily distributed within populations (100%), whereas genetic variation among populations was very low (0.46%) and non-significant, the variance component among populations was negative (Vb = −0.00945). In statistics, a negative variance component is a common phenomenon that typically indicates that the genetic variation at this level is not significantly different from random error, meaning there is no real genetic differentiation among populations. Therefore, its contribution rate was recorded as 0% in the presentation of variance distribution proportions. Mantel correlation analysis revealed no significant correlation between genetic distance and geographic distance (*r* = −0.5948, *p* = 0.9583).

### 3.4. Population Dynamics Analysis

To examine whether populations of *D. maroccanus* conform to neutral evolution and to infer their population history dynamics, Tajima’s D and Fu’s Fs neutrality tests were conducted on four geographical populations (Table 5). Tajima’s D values were negative across all populations, with the YN population reaching statistical significance (−2.15611, *p* < 0.05). Fu’s Fs values were consistently negative and significant in all populations (KZ1 population: −3.901, *p* < 0.05; KZ2 population: −8.325, *p* < 0.001; YN population: −2.973, *p* < 0.05; TC population: −19.578, *p* < 0.001).

**Figure 3:**
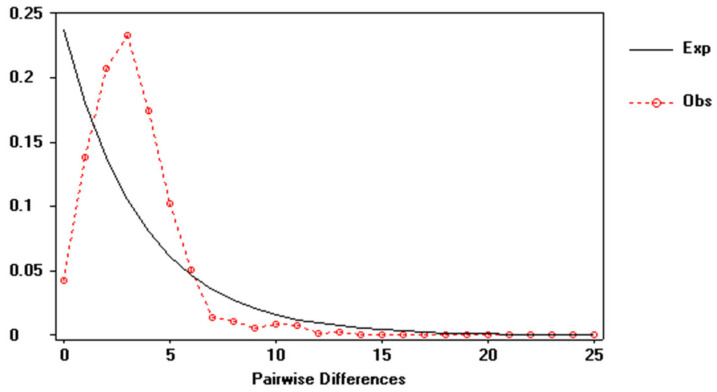
Nucleotide mismatch distribution of combined mitochondrial *COI* and *Cytb* genes of *D. maroccanus* populations along the China–Kazakhstan border. Note: The solid line (Exp) represents the expected distribution under a population expansion model; the dashed line (Obs) represents the observed distribution derived from empirical data.

At the overall level, both the combined Tajima’s D value (−2.45136, *p* < 0.05) and Fu’s Fs value (−44.037, *p* < 0.001) for the four populations were significantly negative, suggesting the possibility of recent population expansion events. Mismatch distribution analysis showed smooth unimodal curves for all populations, with high overlap between the observed (Obs) and expected (Exp) distributions (Figure 3). Mismatch distribution goodness-of-fit tests further showed that KZ1 population (SSD = 0.0028, *p* = 0.91; raggedness = 0.0225, *p* = 0.86), KZ2 population (SSD = 0.00846, *p* = 0.25; raggedness = 0.0650, *p* = 0.13), and YN population (SSD = 0.01121, *p* = 0.12; raggedness = 0.03088, *p* = 0.089) had SSD and raggedness results that were all higher than significance levels, suggesting the mismatch distribution of these populations conforms to the demographic expansion model (Table 6).

## 4. Discussion

*D. maroccanus*, confirmed as a major transboundary migratory pest newly recorded in China in 2025, has emerged in the Tacheng city and the Ili River Basin of Xinjiang, posing a potential challenge to food security, livestock production, and ecological stability in the northwest border region. Understanding its population genetic structure holds important implications for reconstructing dispersal pathways, assessing colonization potential, and developing targeted control strategies. Based on combined mitochondrial *COI* and *Cytb* gene sequences, this study conducted preliminary analyses of genetic diversity, population structure, and demographic history of 74 individuals from four populations along the China–Kazakhstan border, providing preliminary data for the genetic pattern of this invasive species in this region.

The combined mitochondrial *COI* and *Cytb* gene sequences exhibited a significant AT bias (A + T content of 68.1% and C + G content of 31.9%), consistent with typical insect mitochondrial genomes [32,33]. A total of 41 haplotypes were identified among the 74 samples, including seven shared haplotypes and 34 low-frequency exclusive haplotypes. Shared haplotypes are generally considered to be derived from ancestral lineages and tend to be relatively stable, whereas exclusive haplotypes are generally associated with the accumulation of mutations following population adaptation to specific environmental pressures or geographic isolation [34]. In the haplotype network, the dominant shared haplotypes H3 and H6 were widely distributed across all populations, suggesting they may represent ancestral haplotypes with strong persistence and broad adaptability. The structure formed by numerous low-frequency exclusive haplotypes is consistent with patterns observed in other migratory pests, such as *Calliptamus italicus* and *Calliptamus barbarus* [35,36,37]. This pattern suggests that both the potential source (KZ1 and KZ2 populations) and newly established (YN and TC populations) populations may share shared haplotypes, suggesting a common genetic origin and possible rapid expansion across these regions. However, this study is limited by the absence of samples from other potential source regions in Kazakhstan, as well as from neighboring countries such as Kyrgyzstan, Uzbekistan, and Tajikistan. Additionally, incomplete survey data from more distant regions including southern Russia and the Mediterranean coastline mean that these areas cannot be ruled out as potential invasion source populations.

Among all haplotypes, the YN population exhibited the highest number of exclusive haplotypes (18), far exceeding that of the other populations. This finding is somewhat analogous to the unique genetic characteristics reported for populations in the Iberian Peninsula by González-Serna et al., in which “higher genetic distinctiveness in peripheral populations” was also observed [17]. However, the underlying mechanisms differ: the Iberian populations are attributed to long-term geographic isolation and the glacial refuge effect, whereas the high haplotype uniqueness observed in the YN population may be explained by two alternative scenarios: (i) if this species is a newly recorded species that recently entered China from overseas, the high haplotype diversity in the YN population may reflect the accumulation of multiple independent introduction events combined with local environmental adaptation; (ii) alternatively, if the species has long existed in transboundary regions of Central Asian countries but was insufficiently recorded due to historical survey gaps, then the high uniqueness in the YN population actually reflects long-standing local adaptive differentiation of this species in China’s border regions. These two scenarios differ fundamentally in terms of temporal scale and formation mechanisms, and our current mitochondrial markers alone cannot definitively distinguish between them. In terms of specimen evidence, we reviewed locust specimen records from relevant regions in China over the past two decades and found no records of this species. This result supports, to some extent, the possibility that this species was recorded relatively recently. Future work combining nuclear genome markers and more extensive cross-border sampling will help distinguish between these two possibilities.

Genetic diversity directly or indirectly influences the process by which invasive species establish populations in their host regions. The four geographic populations of *D. maroccanus* exhibited a characteristic pattern of high haplotype diversity (*H*d = 0.957) and low nucleotide diversity (*π* = 0.00291), with similar genetic structures across all populations. The nucleotide diversity values of Orthoptera mtDNA genes typically span a wide range, including published data on: *Calliptamus italicus* (*π* = 0.0022) [36], *Calliptamus barbarus* (Hd = 0.987, π = 0.0084) [38], *Oedaleus infernalis* (*π* = 0.0036 ± 0.0004) [39], and the invasive species *Tuta absoluta* (*π* = 0.00005) [20]. In comparison, the nucleotide diversity of *D. maroccanus* (*π* = 0.00291) falls at a relatively moderate-to-low level. This “high *H*d, low *π*” pattern observed in *D. maroccanus* is similar to the genetic characteristics observed in some newly invasive species reported in the literature, i.e., genetic diversity changes may accompany the invasion process of invasive species [40]. Such patterns may have multiple explanatory pathways: it could arise from recent introduction events or reflect the genetic characteristics of a long-standing transboundary population. Although founder effects are often assumed to constrain the adaptive potential of invasive insects, they may, in some cases, actually facilitate population establishment and spread [41]. For example, *Linepithema humile* achieved rapid expansion in North America despite reduced genetic diversity during invasion [42]. This suggests that high levels of genetic diversity are not a prerequisite for successful invasion; in some cases, reduced genetic diversity may even facilitate the establishment and spread of invasive species [43,44]. Therefore, given that *D. maroccanus* has been found in China for only the second year and has low genetic diversity, the potential risks of its colonization and spread should be given serious attention. It is recommended that relevant Chinese authorities strengthen surveillance of existing *D. maroccanus* populations and remain vigilant for potential new introduction events. Among the four populations examined, the YN population exhibited the highest number of polymorphic sites (*S* = 40), haplotypes (*h* = 23), and haplotype diversity (*H*d = 0.986). This pattern indicates that the YN population not only retains shared ancestral haplotypes but may also harbor newly accumulated genetic variation associated with adaptation to local environmental conditions, initially suggesting that it possesses considerable evolutionary potential and dispersal capacity. It is important to emphasize that the large sample size of the YN population may affect the detection probability of rare haplotypes, and this conclusion still needs further verification with subsequent nuclear gene data.

We further evaluated population genetic differentiation indices. Our results revealed that *F*st values between populations ranged from −0.02553 to 0.01990, all well below the threshold for significant differentiation (*F*st < 0.05), where population pairs KZ1 and KZ2 (*F*st = −0.02553), KZ1 and YN (*F*st = −0.00642), and KZ2 and YN (*F*st = −0.01828) showed negative *F*st values indicating no actual genetic differentiation; remaining population pairs had *F*st values approaching zero. Gene flow estimates (*N*m) exceeded 6.25 across all population pairs, with the highest *N*m value observed between YN and TC (*N*m = 43.08). The genetic differentiation level of this species is similar to the results reported by González-Serna et al. [17,45] who used RAD-seq genomic methods to study populations in the Iberian Peninsula (Mean *F*st = 0.067, range = 0.051–0.102), both exhibiting low genetic differentiation. In the same study, this species was also compared with the narrowly distributed congeneric species *D. crassiusculus* (Mean *F*st = 0.129, range = 0.033–0.237), revealing that the latter exhibited significantly higher genetic differentiation. This difference may be associated with certain differences in species dispersal capacity and gene flow patterns. At the same time, low Fst values and high Nm differentiation levels suggest that populations may have high genetic similarity. This genetic similarity exhibits a certain “genetic homogenization” characteristic at the mitochondrial level [46,47]. Nevertheless, we should remain vigilant that a highly uniform genetic background may facilitate synchronized and rapid adaptive evolution in response to environmental changes and pesticide exposure, thereby elevating the risk of population outbreaks and resistance development [42,48]. AMOVA analysis further revealed that genetic variation was distributed almost entirely within populations, while the contribution of between-population variation was negligible. Additionally, the haplotype network showed no distinct clustering corresponding to geographic populations, and the Mantel test detected no significant correlation between geographic and genetic distance (*r* = −0.5948, *p* > 0.05). All above evidence indicates that within the study area, *D. maroccanus* populations along the China–Kazakhstan border may experience frequent gene flow and have not yet formed significant geographic genetic structure. However, this pattern may result from migration promoting gene flow, recent common ancestral effects, recent expansion events, insufficient resolution of mitochondrial markers, or a combination of these factors, and future studies should integrate multi-locus genetic data for comprehensive evaluation.

Neutrality tests revealed significantly negative Fu’s Fs values across all populations (*p* < 0.05–0.001), and the overall Tajima’s D was also significantly negative (−2.45136, *p* < 0.05). Combined with the unimodal pattern shown in the nucleotide mismatch distribution, these results are consistent with the hypothesis that the four populations have may undergone recent expansion events [49]. Mismatch distribution goodness-of-fit tests further supported this inference: the indicators for KZ1 population (SSD = 0.0028, *p* = 0.91; raggedness = 0.0225, *p* = 0.86), KZ2 population (SSD = 0.00846, *p* = 0.25; raggedness = 0.0650, *p* = 0.13) and YN population (SSD = 0.01121, *p* = 0.12; raggedness = 0.03088, *p* = 0.089) were not significantly different from the sudden expansion model. Overall, SSD and raggedness test results were generally consistent with the direction of expansion signals indicated by neutrality tests and unimodal mismatch distribution morphology, further enhancing the credibility of the explanation that “the four populations may have recently experienced expansion.” Regarding the intensity of expansion signals, there were differences among populations. Among them, the fitting effect of SSD and raggedness for KZ1 population was the best among the four populations, combined with its significantly negative Fu’s Fs in neutrality test, suggesting that the genetic pattern of this population may be related to recent expansion. The statistical significance of YN population neutrality tests was the strongest, but its SSD and raggedness goodness-of-fit were weaker than KZ1; combined with the characteristic of high-density distribution in the wild, it indicates the importance of this population in the current diffusion dynamics. For the TC population, neutrality test showed that Fu’s Fs was significantly negative, but Tajima’s D did not reach the level of significance, so the statistical evidence for the expansion signal was relatively weak. Field observations showed that TC population had the lowest density and smallest outbreak scale. What needs to be alert is that a small number of individuals in the early invasion stage does not necessarily indicate population establishment failure [41]. The KZ2 population showed a significantly negative Fu’s Fs in neutrality test, Tajima’s D did not significantly deviate, and its SSD and raggedness both matched the expansion model, but the goodness-of-fit was inferior to KZ1. Based on current results, although KZ1 and KZ2 populations in Kazakhstan also showed signals of demographic expansion, their genetic diversity indices (*S*, *H*d, *π*) were lower than those of the YN population. This suggests that KZ1 and KZ2 populations may not represent the original, highly diverse source populations of the species. Instead, all four populations may have originated from a single, as-yet-unsampled source population with higher genetic diversity than those included in this study.

Mismatch distribution analysis results indicated that *D. maroccanus* may have recently undergone an expansion event. González-Serna et al. [17] employed RAD-seq genomic methodology to investigate the population history of *D. maroccanus* from the Iberian Peninsula, finding that the species experienced a transition from glacial bottleneck to warm-period population expansion during the early Holocene, suggesting that warm climate conditions favor its population growth. Although this study did not estimate specific expansion time, the combined results from existing literature suggest that the dispersal of *D. maroccanus* may be associated with its adaptability to climate change, which requires verification through more spatiotemporal data. Therefore, the relationship between recent population dispersal and ongoing climate warming in this species warrants further investigation in future studies.

Given that *D. maroccanus* is currently in the early stages of establishment in China and has not yet completed a full generation turnover and reproductive cycle, the dynamic changes of its population genetic characteristics require long-term monitoring. This study investigated the genetic diversity and population structure of *D. maroccanus*, which offers certain reference value for formulating effective control strategies. Nonetheless, the current findings offer important insights for prevention and control practices. First, the cross-border monitoring network should be strengthened to systematically collect overseas population samples, covering broader range of habitats and geographic areas to trace true sources and dispersal pathways. Second, integrating genetic data with meteorological and habitat information can help construct precise dispersal routes and predictive models to enhance early warning capacities. Third, incorporating genetic dynamic tracking indicators into joint prevention and control mechanisms would provide a scientific basis for regional disaster prevention and mitigation.

## 5. Conclusions

This study provides the first systematic analysis of genetic diversity, population structure, and demographic history of four geographical populations of *D. maroccanus* along the China–Kazakhstan border based on combined mitochondrial *COI* and *Cytb* gene sequences. The results reveal a characteristic genetic pattern of high haplotype diversity coupled with low nucleotide diversity in the invasive populations. Genetic differentiation among populations is extremely low, and gene flow is high. In combination with the radiation-dispersal pattern observed in the haplotype network and the non-significant Mantel test results, these findings indicate the possible absence of a clear geographic population structure in this species. Furthermore, all populations exhibit significantly negative Fu’s Fs values. Combined with the smooth single-peak nucleotide mismatch distribution and non-significant goodness-of-fit test results for the KZ1, KZ2, and YN populations, these results suggest that this species has recently undergone a population expansion event within the sampling range. In summary, based on combined mitochondrial *COI* and *Cytb* gene sequences, this study provides preliminary information for understanding the genetic background of *D. maroccanus* along the Xinjiang border in China and offers preliminary reference for formulating cross-border monitoring and prevention strategies.

## Figures and Tables

**Table 1:** Sampling information for *D. maroccanus*.

Collection Sites	Population Code	Numbers	Collection Time
Yenbekshy Kazakh District, Almaty Region, Kazakhstan	KZ1	12	June 2025
Ile District, Almaty Region, Kazakhstan	KZ2	16	June 2025
Tacheng City, Xinjiang, China	TC	19	June 2025
Yining City, Xinjiang, China	YN	27	July 2025

Note: This table summarizes the collection details for all samples included in this study.

**Table 2:** Genetic diversity and haplotype distribution of *COI* and *Cytb* genes in *D. maroccanus* populations along the China–Kazakhstan border areas.

Population	*n*	*S*	*h*	*H*d	*π*	*K*
KZ1	12	15	9	0.909	0.00204	3.09091
KZ2	16	14	12	0.950	0.00160	2.43333
TC	19	10	9	0.906	0.00149	2.26901
YN	27	40	23	0.986	0.00291	4.41595
Total	74	60	41	0.957	0.00212	3.22399

Note: *n* = number of sequences; *S* = number of segregating sites; *h* = number of haplotypes; *H*d = haplotype diversity; *π* = nucleotide diversity; *K* = average number of nucleotide differences.

**Table 3:** Pairwise *F*st (below diagonal) and *N*m (above diagonal) values of combined mitochondrial *COI* and *Cytb* genes of *D. maroccanus* populations along the China–Kazakhstan border.

	KZ1	KZ2	TC
KZ1		N/A	12.31
KZ2	−0.02553		18.74
TC	0.01990	0.01316	
YN	−0.00642	−0.01828	0.00577

Note: Negative *F*st values indicate no genetic differentiation between populations. Because *N*m = (1 − *F*st)/(4 × *F*st) becomes mathematically undefined when *F*st ≤ 0, Nm is marked as “N/A”.

**Table 4:** AMOVA of combined mitochondrial *COI* and *Cytb* genes of *D. maroccanus* populations along the China–Kazakhstan border.

Source of Variation	d.f.	Sum of Squares	Variance Components	Percentage of Variation
Among groups	1	1.171	0.00747 Va	0.46%
Among populations within groups	2	2.890	−0.00945 Vb	0%
Within populations	70	113.078	1.61541 Vc	100%
Total variance	73	117.676	1.613 43	100.00%

Note: Negative between-population variance indicates no actual genetic differentiation and was treated as zero in variance proportion calculations.

**Table 5:** Neutrality tests of combined mitochondrial *COI* and *Cytb* genes of *D. maroccanus* populations along the China–Kazakhstan border.

Population	Tajima’s D	*p*	Fu’s Fs	*p*
KZ1	−1.62175	*p* > 0.05	−3.901	*p* < 0.05
KZ2	−1.64597	*p* > 0.05	−8.325	*p* < 0.001
TC	−0.73397	*p* > 0.05	−2.973	*p* < 0.05
YN	−2.15611	*p* < 0.05	−19.578	*p* < 0.001
Total	−2.45136	*p* < 0.01	−44.037	*p* < 0.001

**Table 6:** Mismatch distribution goodness-of-fit tests of combined mitochondrial *COI* and *Cytb* genes of *D. maroccanus* populations along the China–Kazakhstan border.

Population	SSD	*p*	Raggedness	*p*
KZ1	0.00280	*p* = 0.91	0.0225	*p* = 0.86
KZ2	0.00846	*p* = 0.25	0.0650	*p* = 0.13
TC	N/A	N/A	N/A	N/A
YN	0.01121	*p* = 0.12	0.03088	*p* = 0.089

Note: The least-square fitting for the TC population did not converge after 2000 steps, so SSD and raggedness could not be reliably estimated and are therefore indicated by “N/A” in the table.

## Data Availability

The *Cytb* and *COI* sequences of *D. maroccanus* supporting this study have been deposited in GenBank under accession numbers PZ436518-PZ436591 and PZ451009-PZ451082, with public release scheduled for August 25.
